# Spectroscopic and Molecular Docking Studies of the *in Vitro* Interaction between Puerarin and Cytochrome P450

**DOI:** 10.3390/molecules19044760

**Published:** 2014-04-16

**Authors:** Jiangjuan Shao, Jianwei Chen, Tingting Li, Xiaoli Zhao

**Affiliations:** College of Pharmacy, Nanjing University of Chinese Medicine, Xianlin Avenue No. 138, Nanjing 210023, China; E-Mails: chenjw695@126.com (J.C.); ttli734493813@163.com (T.L.); xlee_zhao@163.com (X.Z.)

**Keywords:** puerarin, cytochrome P450, spectroscopy, molecular docking

## Abstract

Puerarin, an isoflavone glycoside extracted from *Pueraria* plants, has various medical functions. Cytochrome P450s (CYPs) are crucial phase I metabolizing enzymes, which have been spotlighted for their effects on drug metabolism. The interaction between puerarin and CYPs (CYP1A2 and CYP2D6) was investigated by fluorescence, UV-Vis and circular dichroism spectroscopies, as well as molecular docking, to explore the underlying mechanism under simulated physiological conditions. The molecular docking results indicated that puerarin interacted with CYPs mainly by hydrophobic force and hydrogen bonding. The fluorescences of CYPs were quenched statically. Binding constants (K_a_) and number of binding sites (n) at different temperatures were calculated, with the results being consistent with those of molecular docking. At the same temperature, puerarin bound to CYP1A2 more weakly than it did to CYP2D6. UV-Vis and circular dichroism spectroscopies confirmed the micro-environmental and conformational changes of CYP1A2 and CYP2D6. The findings provide reliable evidence for clarifying the structures and functions of CYPs.

## 1. Introduction

Puerarin (4′,7-dihydroxy-8-β-D-glucose isoflavone), an isoflavone glycoside extracted from *Pueraria* plants, can lower blood pressure, resist oxidation, arrhythmia and inflammation, as well as inhibit tumor cell proliferation [1,2,3,4]. Cytochrome P450s (CYPs), which are crucial phase I metabolizing enzymes, have been spotlighted for their effects on drug metabolism. Most drugs are detoxified via CYP-dependent pathways. Particularly, approximately 90% of drugs are metabolized by CYP1A2, CYP2C9, CYP2C19, CYP2D6 and CYP3A4 [5], which may be interrupted in case their activities are induced or inhibited [6].

Recently, the interactions between small molecules (puerarin herein) of Traditional Chinese Medicine (TCM) and biomacromolecules (CYPs herein) have been extensively studied [7,8]. Unraveling the interactions on the molecular level is conducive to understanding the pharmacological actions of drugs *in vivo*, as well as designing and screening novel drugs with lower toxicities and side effects. Wang *et al*. studied the effect of puerarin on different cytochrome P450 activities by probes. Their conclusions proved that puerarin had no significant effects on the activities of CYP2C9, CYP2C19 and CYP3A4, but remarkably inhibited the activity of CYP1A2 and CYP2D6 in a dose-dependent fashion [9]. Therefore, the interactions between them were investigated herein by fluorescence, UV-Vis and circular dichroism (CD) spectroscopies, as well as molecular docking, aiming to provide evidence for studying the understanding the interactions between CYPs, this and other drugs.

## 2. Results and Discussion

### 2.1. Fluorescence Spectroscopy

#### 2.1.1. Quenching of CYPs Fluorescence by Puerarin

**Figure 1:**
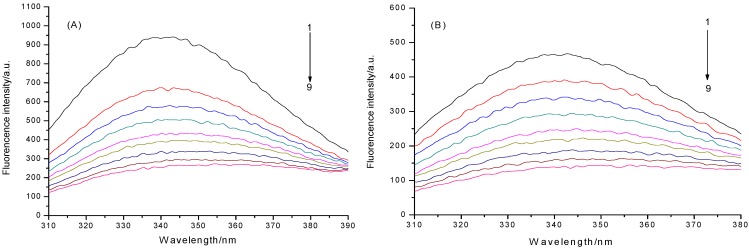
Fluorescence spectra of CYPs in the presence of various concentrations of puerarin (T = 303 K, concentrations of puerarin (1→9): 0, 0.5, 1, 1.5, 2, 2.5, 3, 3.5 and 4 × 10^−5^ mol L^−1^). (**A**) puerarin-CYP1A2; (**B**) puerarin-CYP2D6.

Tryptophan (Trp), tyrosine (Tyr) and phenylalanine (Phe) residues in proteins have a fluorescence intensity ratio of 100:9:0.5. Therefore, protein fluorescence mainly originates from Trp residues, and by measuring it we can predict the conformational changes before and after interacting with drugs. In this study, the quenching of CYPs’ fluorescence by puerarin was studied when excited at 280 nm at 293 K or 303 K. The results are shown in Figure 1. Although the fluorescence intensity of CYP1A2 was higher than that of CYP2D6, they were both quenched by puerarin, accompanied by maximum emission shifts, suggesting puerarin bound to the two CYPs, quenched their endogenous fluorescences and altered the microenvironment of emitting groups.

#### 2.1.2. Fluorescence Quenching Mechanism

Quenching refers to any process which decreases the fluorescence intensity of a given substance. A variety of processes, such as excited state reactions, energy transfer, complex formation and collisional quenching, can result in quenching. The fluorescence of protein is quenched by drug molecules both dynamically and statically, and the quenching efficiency follows the Stern–Volmer relationship [10]:

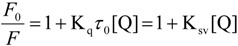
(1)
where F_0_ is the intensity without a quencher, K_q_ is the rate constant of bimolecular quenching, K_sv_ is the dynamic quenching constant, τ_0_ is the average lifetime of the emissive molecule without a quencher (biomacromolecules: 10^−8^ s), and [Q] is the concentration of the quencher. Plotting F_0_/F *vs.* [Q] indicates the types of quenching, *i.e.*, a straight line for individual static or dynamic quenching, and an upward curve for mixed types [11].

**Figure 2:**
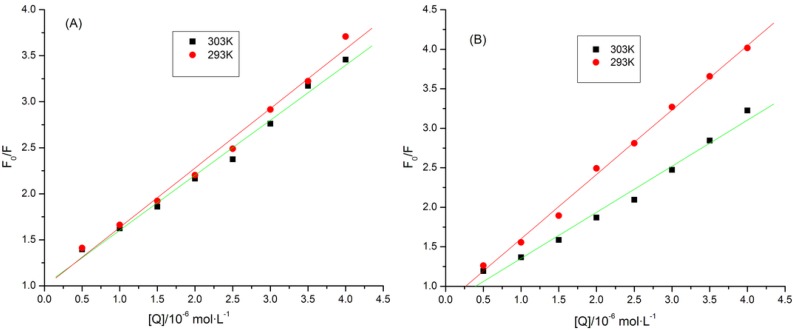
Stern-Volmer plots for the quenching of CYPs by puerarin at different temperatures. (**A**) puerarin-CYP1A2; (**B**) puerarin-CYP2D6.

Figure 2 shows good linear relationships, with the slope decreasing with rising temperature. Meanwhile, the quenching parameters are listed in (Table 1). For instance, the quenching rate constants of puerarin-CYP1A2 at 303 K and 293 K were 5.98 × 10^12^ and 6.46 × 10^12^ L·mol^−1^·s^−1^ respectively, which were higher than the maximum diffusion constant of collisional quenching (2 × 10^1^^0^ L·mol^−1^·s^−1^). Besides, the quenching rate constant reduced with increasing temperature, suggesting that puerarin quenched the fluorescence of CYP1A2 statically instead of dynamically. Likewise, puerarin induced static quenching by forming a complex with CYP2D6.

**Table 1:** Stern-Volmer quenching constants of the systems of puerarin-CYPs at different temperatures.

System	T (K)	K_sv_ (10^4^ L·mol^−1^)	K_q_ (10^12^ L·mol^−1^·s^−1^)
Puerarin-CYP1A2	293	6.46	6.46
	303	5.98	5.98
Puerarin-CYP2D6	293	5.83	5.83
	303	7.86	7.86

#### 2.1.3. Binding Constant K_a_ and Number of Binding Site n

When drug molecules independently bind to a suitable site in a protein, there is an equilibrium between interacting and non-interacting molecules [12]:

lg[(F_0_ - F)/F] = lgK_a_ + nlg[Q] (2)

The apparent binding constant K_a_ and binding site n were calculated by plotting lg [(F_0_ − F)/F] *vs.* lg[Q] at different temperatures [13,14,15] (Table 2).

**Table 2:** Binding constants K_a_ and numbers of binding sites n.

System	T (K)	K_a_ (10^5^ L mol^−1^)	n
Puerarin-CYP1A2	293	0.19	0.89
	303	0.23	0.90
Puerarin-CYP2D6	293	3.39	1.19
	303	6.56	1.21

The stoichiometries between puerarin and CYPs were close to 1:1, and K_a_ increased with rising temperature_,_ which may be attributed to the conformational changes of CYP1A2 and CYP2D6 at various temperatures. Puerarin bound to CYP1A2 more weakly than to CYP2D6, as evidenced by the lower K_a_ and stoichiometry.

#### 2.1.4. Thermodynamic Parameters and Interaction Force Types

Drugs commonly interact with biomacromolecules via hydrogen bonding, hydrophobic interactions, van der Waals force and electrostatic attraction, *etc.* The enthalpy change (ΔH) and entropy change (ΔS) during binding can be considered constants at an almost invariable temperature, which can be calculated by the van’t Hoff equation lnKa = −ΔH/RT + ΔS/R and binding equation ΔG = ΔH − TΔS. Thermodynamic parameters were calculated by the binding constants in Table 2 (Table 3).

**Table 3:** Thermodynamic parameters for the associations of puerarin with CYPs.

System	T (K)	ΔH (kJ mol^−1^)	ΔS (J mol^−1^·K^−1^)	ΔG (kJ mol^−1^)
Puerarin-CYP1A2	293	28.78	180.01	−23.96
	303	28.78	180.01	−25.76
Puerarin-CYP2D6	293	95.60	432.17	−31.03
	303	95.60	432.17	−35.35

Ross *et al.* classified the interaction forces between drugs and protein by ΔH and ΔS into hydrophobic interaction (ΔH > 0, ΔS > 0), van der Waals force (ΔH < 0, ΔS < 0) and electrostatic attraction (ΔH < 0, ΔS > 0) [16].

Since the ΔG values of the two systems were lower than zero, puerarin interacted with CYPs following thermodynamically spontaneous reactions. Moreover, the positive results of ΔH and ΔS indicate puerarin bound to the two CYPs by hydrophobic forces. Puerarin, as a small hydrophobic molecule, intercalated the hydrophobic regions inside CYPs. In addition, hydrogen bonding cannot be ruled out due to the multiple hydroxyl groups of puerarin.

### 2.2. UV-Vis Spectra

**Figure 3:**
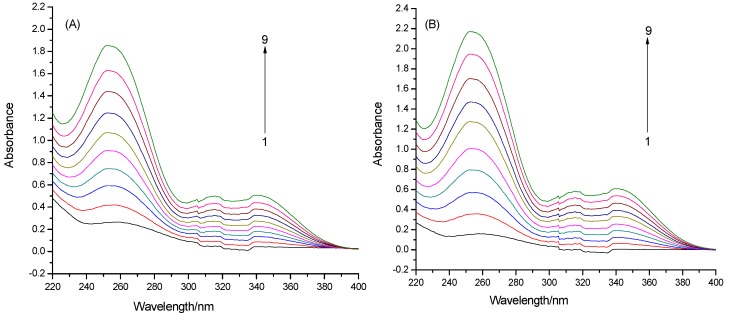
UV-vis spectra of CYPs with different concentrations of puerarin (concentrations of puerarin (1→9): 0, 0.5, 1, 1.5, 2, 2.5, 3, 3.5 and 4 × 10^−5^ mol L^−1^). (**A**) puerarin-CYP1A2; (**B**) puerarin-CYP2D6.

Proteins exhibit UV absorptions owing to Trp (280 nm), Tyr (280 nm) and Phe (257 nm) as well as peptide bonds (225 nm). After adding drugs, the absorptions of protein chromophores are bound to change [17]. As exhibited in Figure 3, CYPs show weak peaks at 257 nm, which can be attributed to the π-π* and n-π* transitions of aromatic heterocycle in Phe. With increasing concentration of puerarin, the peaks hypsochromically shifted to 252 nm and became more intense, revealing that puerarin exposed Phe residues inside CYPs by extending the peptide bonds [18].

### 2.3. CD Spectra

**Figure 4:**
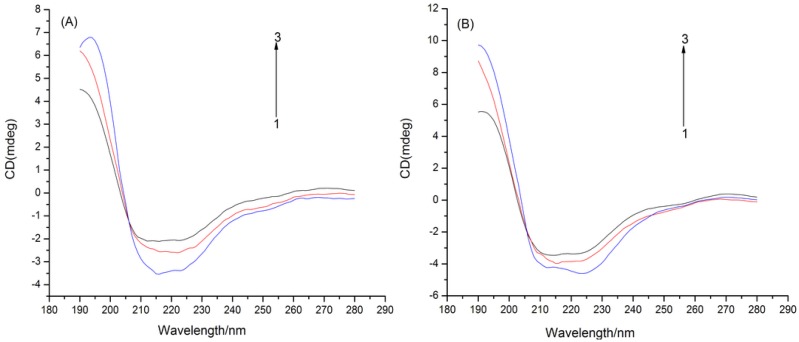
CD spectra of puerarin–CYPs in the absence and presence of increasing amount of puerarin (concentrations of puerarin (1→3): 0, 8 × 10^−4^ and 9 × 10^−4^ mol L^−1^). (**A**) puerarin-CYP1A2; (**B**) puerarin-CYP2D6.

CD spectra were determined to observe the interactions of puerarin with CYPs and corresponding structural changes (Figure 4). CYPs had negative Cotton effects at 212 nm and 223 nm corresponding to α-helix, which were weakened after adding puerarin with slightly varied shapes and positions, suggesting that the secondary structures were altered [19]. With rising puerarin concentration, the α-helix contents dropped probably because hydrophobic force and hydrogen bonding with amino acid residues loosened the secondary structures [20].

### 2.4. Molecular Docking

#### 2.4.1. Molecular Docking of Interactions between Puerarin and CYP1A2

**Figure 5:**
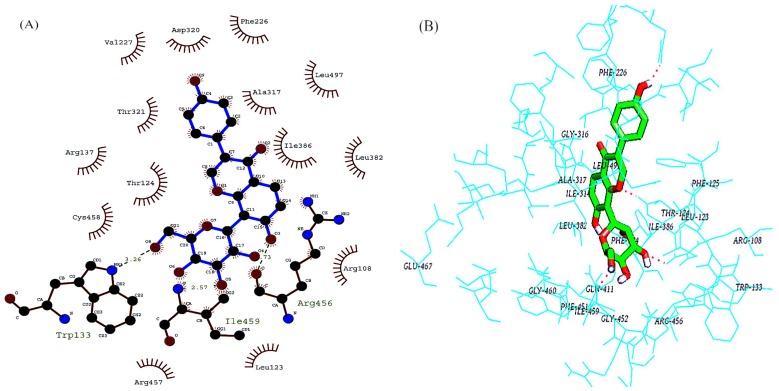
Molecular docking of interactions between puerarin and CYP1A2. (**A**) Ligplus; (**B**) Pymol.

During docking, 50 conformations, one of which had the minimum energy of −7.57 kcal·mol^−1^, were selected. Cluster analysis disclosed that the most probable conformation had the energy of −7.3 kcal·mol^−1^, and 50% docked conformations were included, inferring that the docked structure was reliable and stable. The docking results were also visualized by Ligplus and Autodock (Figure 5).

By using the location of puerarin-containing protein crystal structure as the active site, a three-dimensional box (40 × 40 × 40) encapsulating the protein active center, a hydrophobic cavity, was constructed, mainly including F226, I314, G316 and A317 residues on α−helix as well as F451 and G459 residues on random coils. Puerarin entered the active center by hydrophobic interactions and hydrogen-bound to R456, I459 and W133.

#### 2.4.2. Molecular Docking of Interactions between Puerarin and CYP2D6

**Figure 6:**
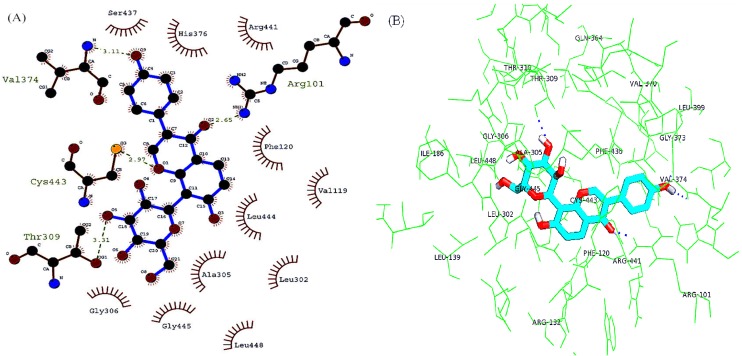
Molecular docking of interactions between puerarin and CYP2D6. (**A**) Ligplus; (**B**) Pymol.

One out of the fifty conformations was of the minimum energy of −7.22 kcal·mol^−1^. Meanwhile, the binding affinity was predicted as ~μM. The results confirm that the docked structure was reliable and stable. The docking results were also visualized by Ligplus and Autodock (Figure 6).

The location of a two drug molecule-containing protein crystal structure was utilized as the active site. The active pocket mainly consisted of three α−helices and random coils that gave rise to hydrophobic grooves, adjoining to which there were THR310, THR309, GLN364, Val370, LEU399, Gly306, Leu448, ALA305, CYS443, Leu302 and phe120, *etc.* Puerarin entered the active center by hydrophobic interactions and hydrogen-bound to V374 andR101. The molecular docking outcomes were consistent with the spectroscopic results by accurately predicting the interactions between puerarin and CYPs.

## 3. Experimental

### 3.1. Apparatus and Reagents

The apparatus used in this study included fluorescence spectrometer (Perkin Elmer LS55, Shelton, WA, USA), UV-Vis spectrometer (Shimadzu UV-2401, Tokyo, Japan), CD spectrometer (JASCO-810, Tokyo, Japan) and pH meter (Leici PHS-25, Shanghai, China).

The reagents included puerarin (standard, National Institutes for Food and Drug Control, Bejing, China), CYP1A2, CYP2D6 (1 × 10^−6^ mol·L^−1^, BD, stored at −80 °C, thawed at room temperature immediately before use), methanol (HPLC grade, Shanghai Lingfeng Chemical Reagent Co., Ltd., Shanghai, China), sodium dihydrogen phosphate, disodium hydrogen phosphate and sodium chloride (analytical grade, Sinopharm Chemical Reagent Co., Ltd., Shanghai, China). Double-distilled water was used throughout the experiments.

### 3.2. Methods

#### 3.2.1. Preparation of Solutions

(1) Preparation of 1×10^−3^ mol·L^−1^ puerarin solution: Puerarin (0.0104 g) was placed in a 25 mL volumetric flask, diluted with HPLC-grade methanol and stored at 4 °C.

(2) Preparation of 0.02 mol·L^−1^ PBS buffer: NaH_2_PO_4_ (0.5928 g) and Na_2_HPO_4_ (5.8019 g) were placed in a 100 mL volumetric flask and diluted with double-distilled water. The solution (10 mL) was then transferred to another 100 mL volumetric flask, diluted with double-distilled water, pH-adjusted to 7.4, and added 0.5844 g NaCl (0.1 mol·L^−1^) to maintain ionic strength.

#### 3.2.2. Fluorescence Spectroscopy

PBS (2 mL) and 10 µL of CYP1A2 (or 3 µL of CYP2D6) were added in a quartz cuvette, to which were added 10 µL aliquots of 1 × 10^−3^ mol·L^−1^ puerarin solution to the final concentrations of 0, 0.5, 1, 1.5, 2, 2.5, 3, 3.5 and 4 × 10^−5^ mol·L^−1^. Being excited at 280 nm, fluorescence spectra were recorded from 290 to 440 nm at 293 K and 303 K respectively.

#### 3.2.3. UV-Vis Spectroscopy

PBS (2 mL) and 10 µL of CYP1A2 (or 10 µL of CYP2D6) were added in a cuvette, to which were added 10 µL aliquots of 1 × 10^−3^ mol·L^−1^ puerarin solution. UV-vis spectra were measured from 220 to 400 nm.

#### 3.2.4. CD Spectroscopy

Various amounts of puerarin solution and 10 µL of CYP1A2 (or 10 µL of CYP2D6) were added in a quartz cell and diluted with PBS buffer to 1000 µL to the final puerarin concentrations of 0, 8 × 10^−4^ and 9 × 10^−4^ mol·L^−1^. By using the corresponding blank solutions as the backgrounds, CD spectra were measured from 190 to 280 nm. Conformations were calculated using the built-in JASCO-810 software.

#### 3.2.5. Molecular Docking

The binding sites and forces between puerarin and CYPs were predicted by molecular docking. The crystal structures of protein receptors human CYP1A2 (entry: 2HI4) and human CYP2D6 (entry: 3TBG) were obtained from the Protein Data Bank. The structure of puerarin was drawn by ChemOffice 11.0, optimized to the structure with lowest energy, and then processed by Autodock through adding hydrogens, calculating electric charges and docking with the protein receptors.

## 4. Conclusions

In summary, the interactions between puerarin and two CYPs were studied by fluorescence, UV-Vis and circular dichroism spectroscopies together with molecular docking. Puerarin quenched the fluorescences of CYP1A2 and CYP2D6 statically, and bound to them mainly by hydrophobic force and hydrogen bonding. At the same temperature, puerarin bound to CYP1A2 more weakly than it did to CYP2D6. Furthermore, puerarin altered the conformations of CYP1A2 and CYP2D6. The findings provide reliable evidence for investigating the interactions between compounds and CYPs as well as for clarifying the structures and functions of CYPs.
